# Molecular dietary analysis of two sympatric felids in the Mountains of Southwest China biodiversity hotspot and conservation implications

**DOI:** 10.1038/srep41909

**Published:** 2017-02-14

**Authors:** Mengyin Xiong, Dajun Wang, Hongliang Bu, Xinning Shao, Dan Zhang, Sheng Li, Rongjiang Wang, Meng Yao

**Affiliations:** 1 School of Life Sciences, Peking University, Beijing 100871, China

## Abstract

Dietary information is lacking in most of small to mid-sized carnivores due to their elusive predatory behaviour and versatile feeding habits. The leopard cat (LPC; *Prionailurus bengalensis*) and the Asiatic golden cat (AGC; *Catopuma temminckii*) are two important yet increasingly endangered carnivore species in the temperate mountain forest ecosystem in Southwest China, a global biodiversity hotspot and a significant reservoir of China’s endemic species. We investigated the vertebrate prey of the two sympatric felids using faecal DNA and a next-generation sequencing (NGS)/metabarcoding approach. Forty vertebrate prey taxa were identified from 93 LPC and 10 AGC faecal samples; 37 taxa were found in the LPC diet, and 20 were detected in the AGC diet. Prey included 27 mammalian taxa, 11 birds, one lizard and one fish, with 73% (29/40) of the taxa assigned to the species level. Rodents and pikas were the most dominant LPC prey categories, whereas rodents, pheasant, fowl and ungulates were the main AGC prey. We also analysed the seasonal and altitudinal variations in the LPC diet. Our results provide the most comprehensive dietary data for these felids and valuable information for their conservation planning.

Knowledge of food web interactions and predator–prey relationships is essential for understanding the ecosystem function of a species and designing conservation strategies^1^. As apex predator populations have declined drastically worldwide as a result of habitat loss and eradication by humans^2^,^3^, small to mid-sized carnivores that often occupied the middle-rank in the predator hierarchy (i.e. mesopredators) have ascended to the top of the food chain and function as apex predators in a range of ecosystems^4^. Mesopredators tend to have more diverse and flexible diets compared to those of large carnivores^5^; hence, their ascendency in the tropic hierarchy can lead to profound changes in the food web structure and affect virtually all other organisms in the ecosystem through cascading effects^4^,^6^. Studies of mesopredator dietary habits and variations provide critical information for identifying the structure and dynamics of trophic interactions, evaluating the ecosystem services provided by predator species, and making informed wildlife management and conservation decisions.

The temperate forests of the Minshan mountains in Sichuan Province are in the northernmost range of the biodiversity hotspot of the Mountains of Southwest China^7^. The original predator assemblage may have included tiger (*Panthera tigris*), leopard (*Panthera pardus*), clouded leopard (*Neofelis nebulosa*), dhole (*Cuon alpinus*) and the wolf (*Canis lupus*), which have all been locally extirpated or are functionally extinct^8^. Mammalian carnivores inhabiting the area include giant pandas (*Ailuropoda melanoleuca*), Asiatic black bears (*Ursus thibetanus*), hog badgers (*Arctonyx collaris*), Chinese ferret badgers (*Melogale moschata*), yellow-throated martens (*Martes flavigula*), Siberian weasels (*Mustela sibirica*), masked palm civets (*Paguma larvata*), leopard cats (LPC; *Prionailurus bengalensis*), and Asiatic golden cats (AGC; *Catopuma temminckii*)^9^,^10^. LPC and AGC are the only known extant members of the family Felidae and key predators in this ecosystem because of their highly carnivorous diets, versatile hunting skills, and adaptability to a wide range of habitat types^11^. LPC and AGC occur in moderate to high population densities relative to other carnivores in this ecosystem based on camera-trapping surveys^9^,^12^ and molecular species identification of faecal samples (see Materials and Methods). However, very little data are available regarding the ecological functions of these cats or their trophic interactions with other species.

LPC is a small felid with an adult body weight range of 1.7–7.1 kg^13^. It is one of the most common wild cats with a wide distribution across Asia and is found in a variety of habitats^14^. The population status of the LPC varies in different regions. The species is listed as least concern on the IUCN Red List^15^, although some local populations and subspecies are endangered^16^. In China, LPC was extensively harvested for fur trade prior to the early 1990 s, and its populations in the South and Southwest China may have suffered significant reductions^17^. Current threats to the species are mainly habitat loss and fragmentation due to anthropogenic land use^8^. No systematic population survey of the LPC has been conducted in China. Although LPC is detected at a relatively high frequency by camera-trapping surveys in the study area^18^, the population status of the species is unclear. Analyses of faeces and stomach contents show that the main LPC prey items are rodents (mostly murids), supplemented with other small mammals, such as squirrels and shrews, birds, reptiles, amphibians and fish, across its range^19^,^20^,^21^,^22^,^23^,^24^,^25^,^26^. Little data are available on their dietary habits in most regions of China.

AGC is a medium-sized felid (adult body weight: 9–16 kg) mostly distributed in Southeast Asia and South and Central China, primarily found in forest habitats^17^,^27^. Due to habitat loss and declining prey abundance, AGC populations are decreasing in most of its range^28^ and this species is listed as near threatened on the IUCN Red List^15^. AGC population status in China is unknown. In the study area, AGC occurs at a much lower density than LPC, according to camera-trapping records^9^. Data on AGC dietary habits are scarce. Morphological analysis of faeces or stomach contents of AGC in Thailand and Malaysia have identified small mammals, such as rodents and ground squirrels, small ungulates, birds, reptiles and amphibians^21^,^29^,^30^. However, AGC diets in China remain uninvestigated.

Conventional dietary analysis methods are primarily based on microscopic examination of hard parts of food remains in faeces or stomach/gut contents. These methods rely highly on undigested portions of prey, expert knowledge of their morphology, are generally time-consuming and often unable to provide precise identification of species^31^. Molecular identification of species based on sequence analysis of polymerase chain reaction (PCR) amplified fragments of a standardised DNA region (i.e. DNA barcoding^32^) from faecal or gut samples provides a reliable and effective alternative to dietary analysis in a wide range of species. This type of analysis requires no *a priori* information on prey composition, is powerful for resolving closely-related species and highly complex diets, is highly efficient for processing large-scale samples and the results can be rechecked and refined at a later time with updated sequence information^31^,^33^. Recent technological advances in next-generation sequencing (NGS) combined with metabarcoding have made DNA-based dietary analysis even more efficient and affable^34^.

We previously analysed the LPC diet in this area using faecal DNA and a traditional cloning-sequencing approach and identified 16 vertebrate taxa in 25 faecal samples^35^. However, due to the small sample size and low throughput sequencing technique, the results are unlikely to represent the complete dietary profile of the species, nor demonstrate the seasonal or regional variations. The goals of this study were (1) to investigate vertebrate prey compositions of LPC and AGC and analyse potential seasonal and altitudinal differences in the diet in the mountain forests of Southwest China using an NGS and metabarcoding-based approach and (2) to inform conservation planning and management actions based on the dietary data.

## Materials and Methods

### Study site and faecal sample collection

**Figure 1:**
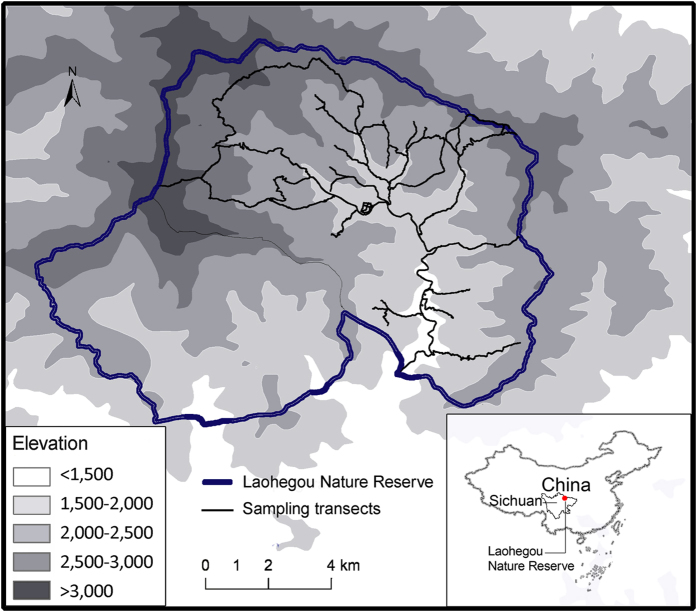
Map of the study site in the Laohegou Nature Reserve for the leopard cat and Asiatic golden cat dietary analyses in northern Sichuan, China. Map was generated in ArcGIS v10.1 (ESRI, Relands, CA, USA) and modified with Adobe Photoshop CS5 software (Adobe Systems Inc., San Jose, CA, USA).

The study site was in the Laohegou Nature Reserve (104°32′42″–104°45′25″ E, 32°25′52″–32°36′22″N, altitude: 1,200–3,500 m; Fig. 1) located in northern Sichuan Province, China. Mean annual rainfall ranges from 950 to 1,300 mm, and mean monthly temperature varies from −4 °C to 12 °C. The Reserve has documented occurrences of 21 mammalian species (excluding small-bodied species, such as rodents, shrews and bats), 188 bird species, 18 reptilian species, 15 amphibian species and five fish species^36^.

We collected faecal samples in the Reserve from March 2013 to November 2014. The majority of samples were collected in spring (March–May) and autumn (September–November). We were unable to obtain samples during the summer season (July–August) or winter months (December–February) due to weather conditions. Mammalian faeces were collected along fixed transects at altitudes of 1,250–3,200 m (Fig. 1) during weekly patrols, submerged in 95% ethanol and stored at −20 °C.

### DNA extraction

We used the 2CTAB/PCI method to extract DNA from faecal samples^37^. We included approximately 100 mg of the external surface in each DNA extraction for molecular identification of species. Faecal samples that were confirmed to be from LPC or AGC were homogenised individually, and 100 mg of the homogenised faeces was used in DNA extraction for the molecular dietary analysis.

### Molecular species identification

We designed the 16S-F/16S-R primer pair to amplify a ~350 bp fragment of the mitochondrial 16 S rRNA gene to unambiguously assign all known predator species in the study area^35^. The LPC and AGC sequences of this fragment differed at 15 nucleotides (GenBank Accession numbers LPC: JN392459.1 and AGC: KP202267.1). The PCR conditions and sequencing of the products were described previously^35^. The species was assigned when faecal DNA amplicons matched the sequences in the GenBank database with 98–100% identity.

We successfully amplified DNA and identified species in 253 (73%) of the 345 field-collected faecal samples, of which 141 were from LPC and 24 were from AGC. Other commonly occurring species included takins (*Budorcas taxicolor, n* = 31), Siberian weasels (*n* = 18), tufted deer (*Elaphodus cephalophus*; *n* = 9), Chinese gorals (*Nemorhaedus griseus*; *n* = 8), wild boars (*Sus scrofa*; *n* = 8), Chinese muntjac (*Muntiacus reevesi*; *n* = 4), Chinese rock squirrels (*Sciurotamias davidianus, n* = 3), hog badgers (*n* = 2), Malayan porcupines (*Hystrix brachyura, n* = 2), Asiatic black bears (*n* = 1), yellow-throated martens (*n* = 1), and complex-toothed flying squirrels (*Trogopterus xanthipe*s, *n* = 1).

We selected LPC and AGC faecal samples to be included in subsequent dietary analyses based on DNA quality and sample location information. Samples that failed to yield sufficient DNA for PCR amplification of food items were excluded. We also eliminated faecal samples collected in close proximity (<50 m), which may represent pellets of a single defecation. A total of 94 LPC and 11 AGC faecal samples met the selection criteria and were included in the NGS run.

### PCR for diet analysis

To amplify DNA of vertebrate species in the faecal DNA extracts by PCR, we used the 12SV5-F/12SV5-R primers targeting a ~100 bp fragment of the mitochondrial 12 S rRNA gene, which has been demonstrated to have high resolution power for identifying the genus and species across most vertebrate taxa^38^. A blocking oligonucleotide (PrioB)^39^ designed to specifically inhibit amplification of the LPC sequences was also included in the PCR. We tested the blocking efficiency of PrioB with AGC samples and found that it also limited amplification of the AGC sequences at high efficiency (Appendix S1). The PrioB sequence diverged from small mammals (pikas, rodents and shrews), ungulates and birds that commonly occur in the study area (Appendix S2) and should not prevent amplification of these vertebrate groups.

PCR amplifications were conducted in a total volume of 20 μL, including 1 × EasyTaq PCR SuperMix (TransGen Biotech, Beijing, China), 0.2 μM 12SV5-F/R primers, 4 μM PrioB, 0.4 mg/mL bovine serum albumin and 20–40 ng DNA extract. The PCR program started with an initial denaturation step of 5 min at 95 °C, followed by 35 cycles of 30 s at 95 °C, 30 s at 60 °C and no elongation. The 12SV5-F and 12SV5-R primers were tagged at the 5′ end with nine nucleotides that started with CC followed by seven variable nucleotides^40^. These tags differed by at least three nucleotides among the tags; hence, allowing a unique tag for each PCR to sort sequences according to samples following NGS. Seven negative PCR controls were also included in the amplifications to check for contamination.

### Next-generation sequencing

PCR products were purified using a PCR purification kit (EasyPure PCR Purification Kit, TransGen Biotech). DNA concentrations were determined by agarose gel electrophoresis and using a NanoDrop 2000 spectrophotometer (ThermoScientific, Waltham, MA, USA) and were mixed in equimolar concentrations. Sequencing was carried out on the Illumina HiSeq 2500 (Illumina Inc., San Diego, CA, USA) at the NGS facility of the Biodynamic Optical Imaging Centre, Peking University, using paired-end sequencing following the manufacturer’s instructions. A total of 100 nucleotides were sequenced on each end of the DNA fragment.

### NGS data analysis

The sequence reads were first checked using the FastQC program to evaluate data quality, and low quality reads were filtered with the NGSQCToolkit v2.33.

We used the *OBITools* program^41^ to analyse the sequence reads. The direct and reverse sequences were aligned using the *illuminapairedend* program, and aligned sequences with a quality score <40 were removed using the *obigrep* program. Sequences with perfectly matched tags and a maximum of two mismatches in primers were identified using the *ngsfilter* program and kept for further analysis. Identical sequences were clustered into unique sequences using the *obiuniq* program. Sequences <80 bp or with a total count in the whole dataset <1,000 were removed using the *obigrep* program. PCR and sequencing errors were detected using the *obiclean* program^42^. A reference database was prepared by extracting vertebrate 12 S gene sequences from GenBank with the 12SV5 primers using the *ecoPCR* program^43^. Taxonomic identification was conducted using the *ecoTag* program^44^ based on sequence similarity in this reference database. A unique taxon was assigned to each sequence. Sequence alignment and automatically assigned taxa were checked manually. We excluded sequences with <94% identity to the query sequence to increase accuracy of the taxonomic assignments. We removed obvious human contaminants, sequences of the two felids and low-frequency sequences (<0.1% or 50 reads in the sample or less than the count of the sequence in the negative control PCRs) that were the likely result of cross-contamination and/or tag jumps^45^. The unique sequences were further collapsed into discrete taxa using a 2% sequence divergence threshold and relative abundance of the sequences. We refined the automatic taxonomic assignments with species distribution data from the study area^14^,^36^,^46^.

We used the following criteria for taxonomic assignments: (1) if the query sequence matched a single locally occurring species in the databases with ≥98% identity, a species was assigned; (2) if the query matched more than one species with ≥98% identity, a species was assigned based on knowledge of the distribution or the lowest taxonomic level that included all of the species was assigned if more than one species occurred locally; and (3) if the query matched the reference sequence with a maximum identity of <98% but ≥94%, the query was assigned to the lowest taxonomic level that included all locally occurring species with the highest identity scores. If a single species showed the highest identity but was not known to inhabit the area, the most closely related local species within the same genus was assigned. Locally occurring species were identified using guides to the mammals^14^ and birds^46^ of China, and a survey of species in the Laohegou Nature Reserve^36^ that included (but was not limited to) mid- to large-sized mammals, birds, amphibians, and fish. To avoid misidentification of taxa due to insufficient local species records, we kept to conservative taxonomic assignments and only excluded species that showed no occurrence in all of Southwest China.

### Diet analysis

The LPC and AGC diets were quantified in two ways:

(1) As a percent frequency of occurrence in faecal samples (%FC):


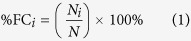


where *N*_*i*_ is the number of faecal samples containing the *i*th food resource, and *N* is the total number of faecal samples containing food;

(2) As a proportion of occurrence of an individual food resource (%TX):


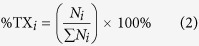


The %TX of all food resources summed to one, but the sum of %FC of the food resources could be larger than one due to the presence of multiple food items in the faecal sample.

Calculation of the dietary parameters including the standardised Levins’ measure of niche breadth (*B*_*A*_), Shannon’s diversity index (*H*), Peilou’s *J* and Pianka’s measure of niche overlap (*O*_*jk*_) followed the equations shown in Appendix S3.

We analyzed the altitudinal variation in the LPC diet by dividing the entire altitudinal range (1250–2300) into four classes: low (<1,500 m), lower-middle (1,500–2,000 m), higher-middle (2,000–2,500 m), and high (>2,500 m). The definition of altitude classes was mainly based on vegetation type, as the distribution of forest types shows a strong altitudinal pattern. Mainly evergreen forests are observed at <1,500 m, evergreen and deciduous mixed forests occur at 1,500–2,500 m, and deciduous and coniferous mixed forests and coniferous forests occur at 2,500–3,200 m. Since vegetation composition and structure also exhibits gradual changes along altitude gradients within each vegetation zone, we further divided the middle zone (1,500–2,500 m) into lower- (1,500–2,000 m) and higher- (2,000–2,500 m) classes to achieve relatively even ranges of the altitude classes.

We used the Wilcoxon signed-rank test to evaluate the seasonal difference of the main food categories in the LPC diet. Statistical significances of altitudinal variation in the LPC diet were assessed for each main prey taxon across the altitude classes with Fisher’s exact tests. We estimated the 95% Wilson score intervals of the frequency of occurrence for the prey orders with the “Hmisc” package^47^ in *R* software^48^.

Statistical analyses were conducted using *R* software^48^. All comparisons were two-tailed, with the significance level set to 0.05.

## Results

### NGS data analysis

The sequencing run generated 4.05 Gb of sequence data (40.1 million raw sequences), which was assembled to produce 18.6 million properly assembled (paired) sequences. After removing low quality sequences, primer or tag sequences and unidentified reads, we collapsed the sequences into 398,588 unique sequences. A further step to eliminate short (<80 bp) or low-occurrence (<1,000) sequences resulted in 638 sequences, which were reduced to 205 sequences after removing PCR and sequencing errors. The high-quality sequences were further reduced by removing low (<94%) identity, low per-sample frequency and non-prey sequences and were collapsed into 40 discrete taxa. Based on the species distribution data, we assigned 29 taxa (73%) to species, four to genera, five to family or subfamily and two to order (Table 1). Of the 94 LPC and 11 AGC PCR products, 93 and 10 produced sequence reads, respectively and were included in the following analyses.

### Dietary composition and diversity

Among the total 40 prey taxa, 37 were consumed by LPC and 20 by AGC, with 17 taxa shared by both species. The number of prey taxa per faecal sample varied from one to seven with a mean of 4.1 (SD 1.7) and 3.6 (SD 1.8) for LPC and AGC, respectively. The LPC diet contained 24 mammalian taxa (six orders), 11 bird taxa (three orders), one lizard and one fish, whereas the AGC diet was comprised of 15 mammalian taxa (six orders) and five bird taxa (two orders) (Table 1). The dietary niche analysis at the taxon level showed that LPC had a narrower diet breadth than that of the AGC, as indicated by Levins’ standardised measure (*B*_*A*_: LPC = 0.27; AGC = 0.64). Dietary diversity of the two species was similar (Shannon diversity index, *H*: LPC = 2.78; AGC = 2.80), and diet evenness was relatively high for both species (Peilou’s *J*: LPC = 0.77; AGC = 0.93).

**Figure 2:**
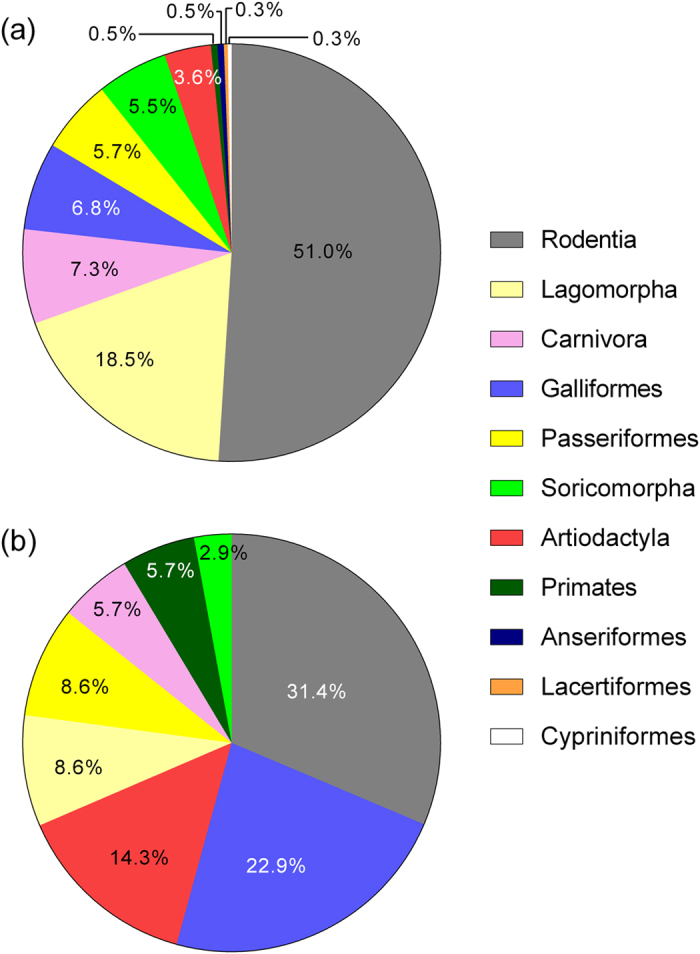
Dietary composition by the prey orders identified in the diets of (**a**) the leopard cat (*n* = 93) and (**b**) Asiatic golden cat (*n* = 10) in northern Sichuan, China. Diet composition by prey order is presented as a proportion of occurrence (%TX).

The most frequently occurring taxon in the LPC diet was pikas (*Ochotona* spp.), which was present in 76% of the faecal samples, followed by several rodents, including the Chinese white-bellied rat (*Niviventer confucianus*; %FC = 62%), South China field mouse (*Apodemus draco*; 44%) and Sichuan Chinese vole (*Eothenomys chinensis*; 40%). Another four taxa were detected in >10% of the LPC samples: undetermined carnivorous species, 29%; large white-bellied rat, *Niviventer excelsior*, 27%; Père David’s vole, *Eothenomys melanogaster*, 19%; and Temminck’s tragopan, *Tragopan temminckii*, 16% (Table 1). Species in the order Rodentia were the most common prey of the LPC when the samples were analysed by the proportion of occurrence of individual prey taxa (%TX = 51%), followed by Lagomorpha (18%) (Fig. 2a).

The most common AGC prey taxa were Temminck’s tragopan (%FC = 60%), the Chinese bamboo rat (*Rhizomys sinensis*; 40%), and pikas (30%). Other taxa occurring in 10% of the AGC samples included the Chinese white-bellied rat (20%) and undetermined carnivorous species (20%) (Table 1). Species in the orders Rodentia (%TX = 31%) and Galliformes (23%) were the most dominant prey of the AGC by proportion of occurrence (Fig. 2b).

### Seasonal and altitudinal variations in the leopard cat diet

**Figure 3:**
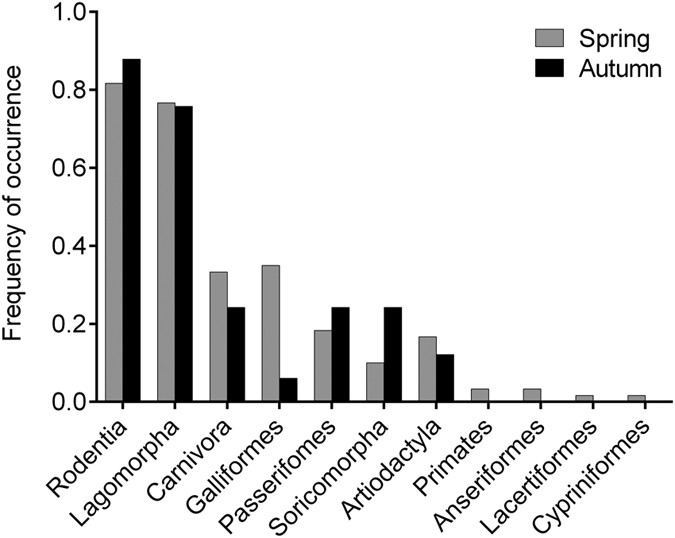
Seasonal variations in diet composition by the prey orders identified in the leopard cat diet in northern Sichuan, China. Spring: *n* = 61; autumn: *n* = 32. Diet composition is presented as a percent frequency of occurrence (%FC).

LPC consumed 33 and 23 prey taxa in spring (*n* = 61 faeces) and autumn (*n* = 32 faeces), respectively. Dietary niche breath in spring (*B*_*A*_ = 0.29) was slightly lower than that in autumn (*B*_*A*_ = 0.41), whereas the Shannon diversity index values were similar in both seasons (*H*: spring = 2.75; autumn = 2.59). The LPC diet composition varied little between spring and autumn, as indicated by very high and significant dietary overlap (*O*_*jk*_ = 0.92, *p* < 0.001) analysed at the taxon level, and similar frequencies of occurrence (%FC) of the seven most common prey orders (Wilcoxon signed-rank test: *Z* = −0.338, *n* = 7, *p* = 0.735; Fig. 3).

**Figure 4:**
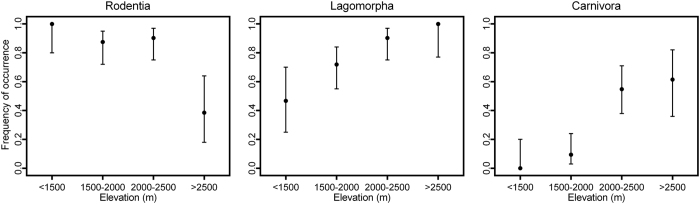
Altitudinal variation in the prey order Rodentia, Lagomorpha, and Carnivora in the leopard cat diet in northern Sichuan, China. <1,500 m: *n* = 15; 1,500–2,000 m: *n* = 32; 2,000–2,500 m: *n* = 31; >2,500 m: *n* = 13. Observed values of frequency of occurrence (%FC) and the 95% Wilson score intervals are shown.

**Figure 5:**
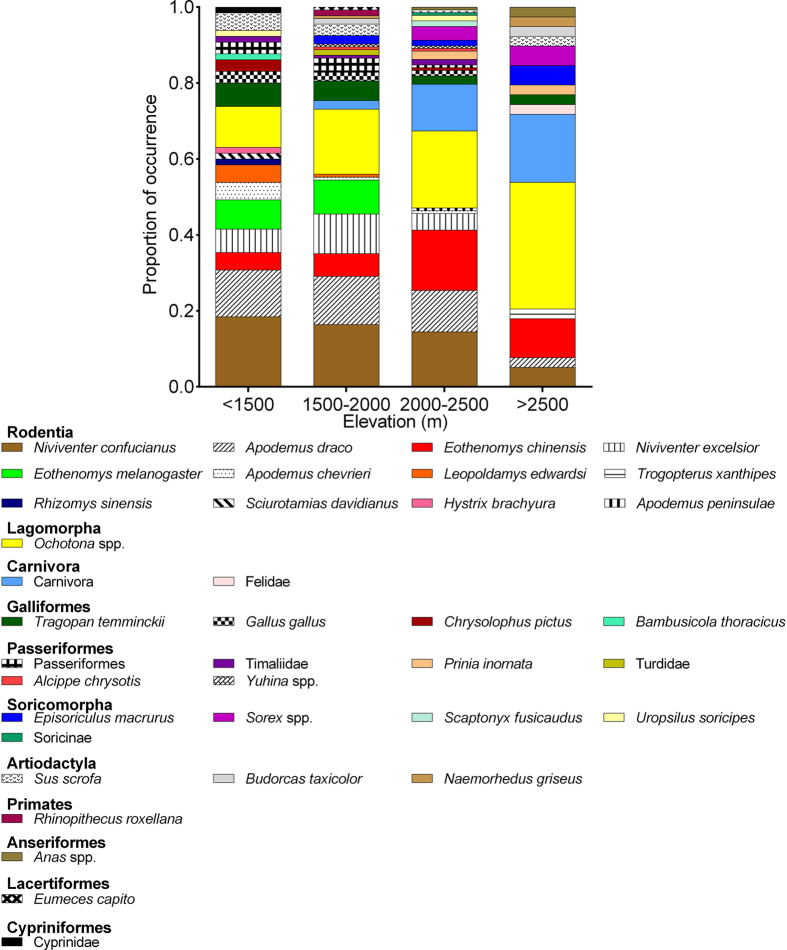
Altitudinal variations in diet composition by the prey taxa identified in the leopard cat diet in northern Sichuan, China. < 1,500 m: *n* = 15; 1,500–2,000 m: *n* = 32; 2,000–2,500 m: *n* = 31; >2,500 m: *n* = 13. Diet composition is presented as a proportion of occurrence (%TX).

The LPC samples were collected at altitudes of 1,250–3,200 m. Species from the seven most common prey orders (Rodentia, Lagomorpha, Carnivora, Galliformes, Passeriformes, Soricomorpha, and Artiodactyla) were detected across the entire altitudinal range (low: <1,500 m; lower-middle: 1,500–2,000 m; higher-middle: 2,000–2,500 m; and high: >2,500 m) (Appendix S4). Fisher’s exact tests showed that the frequency of occurrence (%FC) for Rodentia, Lagomorpha, and Carnivora varied significantly in different altitudinal zones (all *p* < 0.001), whereas altitudinal variations in the occurrence of Galliformes, Passeriformes, Soricomorpha, and Artiodactyla were insignificant. Calculation of 95% confidence intervals for Rodentia, Lagomorpha, and Carnivora indicated distinct distribution patterns for different prey orders (Fig. 4). For example, the frequency of occurrence of rodents was high at low and middle altitudes and reduced by half at altitudes >2,500 m, whereas the occurrence of pikas and carnivores increased with altitude (Fig. 4). At the taxon level, various prey taxa also exhibited different patterns of occurrence (Fig. 5).

## Discussion

### Diets of the two felids

We identified 37 vertebrate taxa in the LPC diet. The main LPC prey categories in the present study generally agree with those of previous studies at the ordinal level but vary in specific species composition. Studies across the LPC range generally show that small rodents, particularly *Rattus* and *Mus* spp., are dominant prey items^20^,^23^,^39^. However, we found that *Niviventer, Apodemus* and *Eothenomys* spp. were the most common rodents in the LPC diet but no *Rattus* or *Mus* spp. were detected. No systematic population survey on small mammals has been conducted in the Laohegou Nature Reserve. Although no *Mus spp.* have been found in the study area, two *Rattus* species, *R. nitidus* and *R. norvegicus* are reported to occur in evergreen broadleaf forests (<1600 m) of the adjacent Tangjiahe Nature Reserve at densities similar to or higher than *Niviventer spp.*^49^, raising questions about the prey selectivity of LPC. Additionally, pikas have been reported as LPC prey in our study area, but not in other regions, even where they are common food of other similar-sized carnivores^50^. Prey composition and availability along altitudinal gradients have been demonstrated to shape the diets of several carnivorous species^51^,^52^. Studies in Tangjiahe Nature Reserve showed that *Niviventer* and *Apodemus* spp. were the most abundant groups below 2,500 m in various forest types, but their numbers diminished quickly above this altitude; pikas were not detected below 1,800 m, occurred at a low number at 1,800–2,500 m, yet prevailed above 2,500 m in Alpine meadows and scrub habitats^53^. The altitudinal gradient in the occurrence of rodents and pikas is roughly correlated with these distribution data, despite that pikas were also frequent in the LPC diet at mid-elevations (1,500–2,500 m) in our analysis, suggesting a versatile generalist feeding tactic that may be influenced more by prey availability than a specific food preference. The above interpretation is based on the assumption that prey remains in faeces reflect predation occurring in the same altitudinal zone. However, LPC can move across large distances in short periods of time^21^, potentially compromising the association between faecal sampling and predation location. The impacts of altitude on LPC prey composition bear further investigation. Additionally, we detected several ungulates in the LPC diet. Thus, this small cat may possibly catch young wild boars and Chinese gorals, which are smaller-bodied ungulates, whereas the occurrence of takins in the diet may most likely be explained as scavenging behaviour. Our analyses also show that LPC consume a wide variety of birds, from small passerines to large ground-dwelling pheasants, demonstrating the predator’s versatile hunting skills.

We identified 20 vertebrate taxa in the AGC diet, representing the most comprehensive dietary analysis reported for this species. Similar to the LPC, AGC also frequently consumed rodents and pikas, but the proportions of larger prey, such as pheasants and ungulates, were higher than those in the LPC diet, possibly reflecting their larger body size compared to that of LPC. Due to the small sample size of AGC, the quantitative dietary analysis results regarding prey diversity remain tentative. AGC appeared to occupy only a portion of the LPC’s range in the study area, as AGC faeces were found only within a relatively narrow altitudinal zone (2,300–2,700 m), whereas LPC faeces were collected throughout the reserve across all altitudes from 1,250 to 3,200 m. Additionally, the AGC population density is likely to be only a fraction of that of LPC in the study area^18^, which is in accordance with the different numbers of faecal samples collected for the two species. Further investigation of the AGC diet is required to better understand this species’ food web hierarchy and niche interactions with other sympatric carnivores.

### Molecular dietary analysis

Most previous studies of the LPC diet were based on microscopic examination of food remains in faeces where generally <10 taxa were identified, often only to the family or higher taxonomic levels^20^,^21^,^23^, demonstrating the superior resolution power of the faecal DNA-based method. The number of vertebrate prey categories identified in the present study (37 taxa) considerably exceeds not only reports based on a traditional cloning-Sanger sequencing approach for the LPC diet in the same area (16 taxa)^35^ and in South Korea (13 taxa)^25^, but also a study based on NGS analysis of LPC faeces collected in northern Pakistan (18 taxa)^50^. Technical advances represented by the NGS approach offer remarkably higher throughput and economy compared to traditional morphological analysis and sequencing methods, demonstrating the high detection sensitivity and assignment power of molecular-metabarcoding/NGS methods. The difference in the number of food taxa between the two NGS-based studies can be explained by the higher species diversity of potential prey in the mountain forests of Southwest China compared to those of northern Pakistan, and the larger sample size of our study (93 vs. 38 faecal samples).

However, species resolution and accuracy using molecular identification are critically dependent on the completeness and quality of the reference databases, and limited coverage of the potential prey can strongly impede species-level identification. We found that the pika sequences in LPC faeces matched the *Ochotona* spp. sequences with 94–99% identity, suggesting that there might be more than one pika species in the diet. Of the eight *Ochotona* spp. potentially present in the study area, including *O. cuzoniae, O. cansus, O. erythrotis, O. gloveri, O. huangensis, O. macrotis, O. thibetana* and *O. thomasi*^14^, only the 12 S rRNA sequence of *O. cuzoniae* is available in the GenBank database. Therefore, we only assigned this taxon to the genus. Pikas were the most frequently occurring food item at the individual taxon level; yet when analysed at the ordinal level, they showed a lower proportion of occurrence in the diet than rodents due to the lack of species-level resolution within the genus *Ochotona*. One promise of the molecular dietary analysis is that taxonomic assignments can be refined as reference databases improve.

The species resolution of molecular identification approaches is also restricted by the extent of sequence divergence among closely-related species and can show varying degrees of discriminating power for different taxa. The DNA barcode used in our study demonstrated high species resolution in most mammalian groups, yet low power differentiating avian species (particularly babblers and ducks). Limited species resolution can be particularly challenging in fine-scale dietary analysis when the study area has high species diversity, such as ours, where nearly 200 bird species have been recorded^36^. Thus, a combination of DNA barcodes targeting specific food groups has been recommended to improve species-level identification^54^.

### Ecosystem function of mesopredators and conservation implications

Removal of apex predators from an ecosystem can lead to loss of top-down control and subsequent outbreaks of mesopredator populations, an effect known as “mesopredator release^55^”, which may result in declines in prey populations and eventual “ecological meltdown^56^”. Despite overall extirpation of former apex predators in many mountain forests in Southwest China, neither explosive growth of mesopredator populations, nor depletion of the main prey groups have been observed^18^,^36^. Habitat loss and fragmentation, hunting by humans, intra-guild competition and limited prey resources may account for the lack of apparent increase in mesopredators in this ecosystem. Ecological processes in such a complex ecosystem can be multivariate and more intricate, and the dynamics and consequences following natural and human disturbances may not fully agree with predications often deduced from analyses of comparatively simple ecosystems^56^. Therefore, a closer examination of the many environmental, biological (intraspecific and interspecific) and anthropogenic factors is required in a particular ecosystem to gain a better understanding of the population dynamics and to predict the patterns of ecological response.

Our results indicate that rodents and pikas constitute the majority of the felids’ diets (particularly the LPC) in the forest ecosystem of Southwest China. These small mammals are generally considered pests, as they degrade forests and grasslands and have been targets of control by poisoning and execution in many regions of China^57^,^58^. However, small mammals assist with seed dispersal and improve plant diversity^59^, provide important prey for many carnivores and predatory birds^60^,^61^ and may serve as keystone species to enhance ecosystem functioning^62^,^63^. Pest control by poisoning has been demonstrated to be ineffective and often incurs undesirable consequences on the environment and untargeted species^64^. In contrast, conserving predator communities is an ecologically safe, economic and sustainable strategy to control small mammalian populations and maintain ecosystem equilibrium, stability and resilience^65^.

In conclusion, our results reveal the highly diverse dietary habits and versatile foraging skills of two felids. The other mammalian carnivores (e.g., Asiatic black bears, hog badgers, Chinese ferret badgers, yellow-throated martens, and masked palm civets) in this ecosystem are largely omnivorous, but LPC and AGC are highly carnivorous and may play pivotal roles as predators^66^. Large proportions of the LPC and AGC diets were composed of pikas and rodents, suggesting that these predators play an important role controlling their populations in the mountain forests of Southwest China. Therefore, caution should be taken in pest control as it may pose serious threats to predators and ecosystem sustainability. The faecal DNA and NGS-based approach enabled a very large-scale dietary analysis and fine species resolution. The remarkable taxonomic discriminating power offered by the molecular approach was particularly powerful to disentangle the food web structure in a highly complex ecosystem such as our study area, where closely related and morphologically similar prey species coexist.

## Additional Information

**How to cite this article**: Xiong, M. *et al*. Molecular dietary analysis of two sympatric felids in the Mountains of Southwest China biodiversity hotspot and conservation implications. *Sci. Rep.* **7**, 41909; doi: 10.1038/srep41909 (2017).

**Publisher's note:** Springer Nature remains neutral with regard to jurisdictional claims in published maps and institutional affiliations.

## Supplementary Material

Supplementary Appdendex 1-4

## Figures and Tables

**Table 1:** Prey taxa identified in the diets of the leopard cat (LPC) and Asiatic golden cat (AGC) in northern Sichuan, China.

Accession no.	Putative taxon		No. of occurrence in LPC faeces (*n* = 93)	No. of occurrence in AGC faeces (*n* = 10)	Sequences with maximum identity in GenBank		
	Scientific name	common name			species	Maximum identity	Accession no.
Rodentia							
KU254241- KU254242	*Niviventer confucianus*	Chinese white-bellied rat	58	2	*Niviventer confucianus*	100	KJ152220.1
KU254243- KU254245	*Apodemus draco*	South China field mouse	41	0	*Apodemus draco*	99	HQ333255.1
KU254246- KU254250	*Eothenomys chinensis*	Sichuan Chinese vole	35	1	*Eothenomys chinensis*	96	FJ483847.1
KU254251- KU254254	*Niviventer excelsior*	Large white-bellied rat	25	1	*Niviventer excelsior*	100	JQ927552.1
KU254255- KU254256	*Eothenomys melanogaster*	Père David’s vole	18	0	*Eothenomys melanogaster*	99	KP997311.1
KU254259	*Apodemus chevrieri*	Chevrier’s field mouse	5	0	*Apodemus chevrieri*	100	HQ896683.1
KU254260	*Leopoldamys edwardsi*	Edwards’s long-tailed giant rat	5	0	*Leopoldamys edwardsi*	99	KM434322.1
KU254261	*Trogopterus xanthipes*	Complex-toothed flying squirrel	2	1	*Trogopterus xanthipes*	100	AY227546.1
KU254257- KU254258	*Rhizomys sinensis*	Chinese bamboo rat	1	4	*Rhizomys sinensis*	100	AF326254.1
KU254262	*Sciurotamias davidianus*	Chinese rock squirrel	1	1	*Sciurotamias davidianus*	99	AY227554.1
KU254264	*Apodemus peninsulae*	Korean field mouse	1	0	*Apodemus peninsulae*	98	AJ311142.1
KU254265	*Hystrix brachyura*	Malayan porcupine	1	0	*Hystrix brachyura*	100	AY012117.1
KU254263	*Dremomys pernyi*	Perny’s long-nosed squirrel	0	1	Dremomys rufigenis*	97	KC447304.1
Lagomorpha							
KU254235- KU254240	*Ochotona* spp.	Pikas	71	3	*Ochotona curzoniae*	99	KM225729.1
Carnivora							
KU254278	Carnivora (?)	?	27	2	*Mustela erminea*	94	KM091450.1
KU254279	Felidae (?)	?	1	0	Panthera tigris*	94	EU983280.1
Galliformes							
KU254287	*Tragopan temminckii*	Temminck’s tragopan	15	6	*Tragopan temminckii*	100	FJ752427.1
KU254288	*Gallus gallus*	Red jungle fowl	7	2	*Gallus gallus*	100	KM096864.1
KU254289	*Chrysolophus pictus*	Golden pheasant	3	0	*Chrysolophus pictus*	100	KC749452.1
KU254290	*Bambusicola thoracicus*	Chinese bamboo partridge	1	0	*Bambusicola thoracicus*	98	KC749450.1
Passeriformes							
KU254280	Passeriformes	?	8	1	*Hirundo rustica/Hirundo daurica/Leiothrix lutea*	99	KP148840.1/KJ499911.1/JQ423933.1
KU254283	Timaliidae	Old World babblers	4	0	*Alcippe morrisonia/Garrulax ocellatus*	100	AY167412.1/AF484898.1
KU254281	*Prinia inornata*	Plain prinia	4	1	Prinia subflava*	100	AY136564.1
KU254282	Turdidae	Thrushes	2	1	*Luscinia cyanura/Phoenicurus auroreus*	99	KF997864.1/KF997863.1
KU254284	*Alcippe chrysotis*	Golden-breasted fulvetta	2	0	*Alcippe chrysotis*	100	AF484888.1
KU254285	*Yuhina* spp.	Yuhinas	2	0	*Yuhina nigrimenta*	95	AF376919.1
Soricomorpha							
KU254266	*Episoriculus macrurus*	Long-tailed mountain shrew	7	1	*Episoriculus macrurus*	99	GU981048.1
KU254267	*Sorex* spp.	Shrews	7	0	*Sorex cylindricauda/Sorex minutus*	99	KF696672.1/EF027290.1
KU254268	*Scaptonyx fusicaudus*	Long-tailed mole	3	0	*Scaptonyx fusicaudus*	98	AB106232.1
KU254269	*Uropsilus soricipes*	Chinese shrew mole	3	0	*Uropsilus soricipes*	100	JQ658979.1
KU254270	Soricinae	Red-toothed shrews	1	0	Cryptotis parva*/Blarina brevicauda*	96	JN393209.1/S73801.1
Artiodactyla							
KU254274	*Sus scrofa*	Wild boar	9	2	*Sus scrofa*	100	KM275217.1
KU254275	*Budorcas taxicolor*	Takin	3	2	*Budorcas taxicolor*	100	FJ006534.1
KU254276	*Naemorhedus griseus*	Chinese goral	2	0	*Naemorhedus griseus*	100	KF500173.1
KU254277	*Moschus berezovskii*	Forest musk deer	0	1	*Moschus berezovskii*	100	EU043465.1
Primates							
KU254271	*Rhinopithecus roxellana*	Golden snub-nosed monkey	2	1	*Rhinopithecus roxellana*	100	JQ821835.1
KU254272- KU254273	*Macaca mulatta*	Rhesus macaque	0	1	*Macaca mulatta*	98	AJ842854.1
Anseriformes							
KU254291	*Anas* spp.	Ducks	2	0	*Anas crecca/Anas platyrhynchos/Anas acuta/Anas poecilorhyncha/Anas clypeata/Anas querquedula*	100	KC466567.1/KJ833587.1/KF312717.1/KF751616.1/KF469288.1/AF173691.1
Lacertiformes							
KU254292	*Plestiodon capito*	Yellow-striped skink	1	0	Plestiodon elegans*	97	KM017746.1
Cypriniformes							
KU254293	Cyprinidae	Carps	1	0	*Ctenopharyngodon idella/Hypophthalmichthys molitrix*	100	JQ231115.1/JQ231114.1

Putative taxa were identified with the aid of species distribution information. Sequences with maximum identity only show locally occurring species; a species is indicated by an asterisk (*) if the species with maximum sequence identity is not known to inhabit the study area. Question marks (?) indicate that taxonomic identification requires further information.

## References

[b1] Sheppard S. K. & Harwood J. D. Advances in molecular ecology: tracking trophic links through predator-prey foodwebs. Funct. Ecol. 19, 751–762 (2005).

[b2] Estes J. A. . Trophic downgrading of planet earth. Science 333, 301–306 (2011). 21764740 10.1126/science.1205106

[b3] Ripple W. J. . Status and ecological effects of the world’s largest carnivores. Science 343, 1241484 (2014). 24408439 10.1126/science.1241484

[b4] Prugh L. R. . The rise of the mesopredator. BioScience 59, 779–791 (2009).

[b5] Roemer G. W., Gompper M. E. & Valkengurgh B. V. The ecological role of the mammalian mesocarnivore. BioScience 59, 165–173 (2009).

[b6] Brashares J. S., Prugh L. R., Stoner C. J. & Epps C. W. In Trophic Cascades: Predators, Prey, and the Changing Dynamics of Nature (eds Terborgh J. & Estes J. A.) (Island Press, 2010).

[b7] Myers N., Mittermeier R. A., Mittermeier C. G., da Fonseca G. A. B. & Kent J. Biodiversity hotspots for conservation priorities. Nature 403, 853–858 (2000). 10706275 10.1038/35002501

[b8] Jutzeler E. . Cat News Special Issue: Cats in China. (2010).

[b9] Li S., McShea W. J., Wang D., Lu Z. & Gu X. Gauging the impact of management expertise on the distribution of large mammals across protected areas. Diversity Distrib. 18, 1166–1176 (2012).

[b10] Li S., Wang D., Bu H., Liu X. & Jin T. Camera-trapping survey on the mammal diversity of Laohegou Nature Reserve, Sichuan Province. Acta Theriol. Sinica. 36, 282–291 (2016).

[b11] Mills L. S., Soule M. E. & Doak D. F. The keystone-species concept in ecology and conservation. BioScience 43, 291–224 (1993).

[b12] Bu H. . Spatial co-occurrence and activity patterns of mesocarnivores in the temperate forest of southwest China. PLoS ONE 11, e0164271 (2016). 27723772 10.1371/journal.pone.0164271 PMC5056745

[b13] Sunquist M. E. & Sunquist F. C. In Handbook of the Mammals of the World. Vol. 1. Carnivores (edsWilson D. E. & Mittermeier R. A.) (Lynx Edicions, 2009).

[b14] Smith A. T. . A Guide to the Mammals of China. (Princeton University Press, 2008).

[b15] IUCN. The IUCN Red List of Threatened Species. Version 2016.1. < www.iucnredlist.org>. Downloaded on 07 July 2016 (2016).

[b16] Sanderson J. *et al. Prionailurus bengalensis*. The IUCN Red List of Threatened Species. Version 2016.1. < www.iucnredlist.org> Downloaded on 07 July 2016 (2016).

[b17] Nowell K. & Jackson P. Wild cats, Status Survey and Conservation Action Plan. (IUCN, Gland, Switzerland, 1996).

[b18] Li S., Wang D., Lu Z. & McShea W. J. Cats living with pandas: the status of wild felids within giant panda range, China. Cat News 52, 20–23 (2010).

[b19] Rabinowitz A. Notes on the behavior and movements of leopard cats, *Felis bengalensis*, in a dry tropical forest mosaic in Thailand. Biotropica 22, 397–403 (1990).

[b20] Fernandez D. A. P. & de Guia A. P. O. Feeding habits of Visayan leopard cats (*Prionailurus bengalensis rabori*) in sugarcane fields of Negros Occidental, Philippines. Asia Life Sciences 20, 143–154 (2011).

[b21] Grassman L. I., Tewes M. E., Silvy N. J. & Kreetiyutanont K. Spatial organization and diet of the leopard cat (*Prionailurus bengalensis*) in north-central Thailand. Journal of Zoology (London) 266, 45–54 (2005).

[b22] Rajaratnam R., Sunquist M., Rajaratnam L. & Ambu L. Diet and habitat selection of the leopard cat (*Prionailurus bengalensis borneoensis*) in an agricultural landscape in Sabah, Malaysian Borneo. J. Trop. Ecol. 23, 209–217 (2007).

[b23] Tatara M. & Doi T. Comparative analyses on food-habits of Japanses marten, Siberian weasel and leopard cat in the Tsushima Islands, Japan. Ecol. Res. 9, 99–107 (1994).

[b24] Watanabe S. Factors affecting the distribution of the leopard cat *Prionailurus bengalensis* on East Asian islands. Mammal Stud. 34, 201–207 (2009).

[b25] Lee O., Lee S., Nam D.-H. & Lee H. Y. Molecular analysis for investigating dietary habits: genetic screening of prey items in scat and stomach contents of leopard cats *Prionailurus bengalensis euptilurus*. Zool. Stud. 52, 45 (2013).

[b26] Lee O., Lee S., Nam D.-H. & Lee H. Y. Food habits of the leopard cat (*Prionailurus bengalensis euptilurus*) in Korea. Mammal Stud. 39, 43–46 (2014).

[b27] Sunquist M. E. & Sunquist F. C. Wild Cats of the World. (University of Chicago Press, 2002).

[b28] Sanderson J. *et al. Catopuma temminckii*. The IUCN Red List of Threatened Species. Version 2016.1 < www.iucnredlist.org> Downloaded on 07 July 2016 (2016).

[b29] Lim B. L. Distribution and food-habits of the Asiatic golden cat (*Catopuma temminckii*) in Peninsular Malaysia. Journal of Wildlife and Parks 20, 43–48 (2002).

[b30] Kawanishi K. & Sunquist M. E. Food habits and activity patterns of the Asiatic golden cat (*Catopuma temminckii*) and dhole (*Cuon alpinus*) in a primary rainforest of Peninsular Malaysia. Mammal Stud. 33, 173–177 (2008).

[b31] Pompanon F. . Who is eating what: diet assessment using next generation sequencing. Mol. Ecol. 21, 1931–1950 (2012). 22171763 10.1111/j.1365-294X.2011.05403.x

[b32] Valentini A., Pompanon F. & Taberlet P. DNA barcoding for ecologists. Trends in Ecology and Evolution 24, 110–117 (2008). 19100655 10.1016/j.tree.2008.09.011

[b33] King R. A., Traugott M. & Symondson W. O. C. Molecular analysis of predation: a review of best practice for DNA-based approaches. Mol. Ecol. 17, 947–963 (2008). 18208490 10.1111/j.1365-294X.2007.03613.x

[b34] Thompson K. A. & Newmaster S. G. Molecular taxonomic tools provide more accurate estimates of species richness at less cost than traditional morphology-based taxonomic practices in a vegetation survey. Biodivers. Conserv. 23, 1411–1424 (2014).

[b35] Xiong M. . Molecular analysis of vertebrates and plants in scats of leopard cats (*Prionailurus bengalensis*) in southwest China. J. Mammal. 97, 1054–1064 (2016).

[b36] Li S. Report on Biodiversity Surveys in the Laohegou Nature Reserve. (2013).

[b37] Vallet D., Petit E. J., Gatti S., Levréro F. & Ménard N. A new 2CTAB/PCI method improves DNA amplification success from faeces of Mediterranean (Barbary macaques) and tropical (lowland gorillas) primates. Conserv. Genet. 9, 677–680 (2007).

[b38] Riaz T. . ecoPrimers: inference of new DNA barcode markers from whole genome sequence analysis. Nucleic Acids Res. 39, e145 (2011). 21930509 10.1093/nar/gkr732 PMC3241669

[b39] Shehzad W. . Carnivore diet analysis based on next-generation sequencing: application to the leopard cat (*Prionailurus bengalensis*) in Pakistan. Mol. Ecol. 21, 1951–1965 (2012). 22250784 10.1111/j.1365-294X.2011.05424.x

[b40] Coissac E. In Data Production and Analysis in Population Genomics (eds Pompanon F. & Bonin A.) (Humana Press, 2012).

[b41] Boyer F. . OBITOOLS: a UNIX-inspired software package for DNA metabarcoding. Mol. Ecol. Resour. 16, 176–182 (2016). 25959493 10.1111/1755-0998.12428

[b42] De Barba M. . DNA metabarcoding multiplexing and validation of data accuracy for diet assessment: application to omnivorous diet. Mol. Ecol. Resour. 14, 306–323 (2014). 24128180 10.1111/1755-0998.12188

[b43] Ficetola G. . An *In silico* approach for the evaluation of DNA barcodes. BMC Genomics 11, 434–443 (2010). 20637073 10.1186/1471-2164-11-434 PMC3091633

[b44] Pegard A. . Universal DNA-based methods for assessing the diet of grazing livestock and wildlife from feces. J. Agric. Food. Chem. 57, 5700–5706 (2009). 19566081 10.1021/jf803680c

[b45] Schnell I. B., Bohmann K. & Gilbert M. T. P. Tag jumps illuminated – reducing sequence-to-sample misidentifications in metabarcoding studies. Mol. Ecol. Resour. 15, 1289–1303 (2015). 25740652 10.1111/1755-0998.12402

[b46] MacKinnon J. & Phillipps K. A Field Guide to the Birds of China. (Oxford University Press, 2000).

[b47] Harrell F. E. Hmisc: Harrell Miscellaneous. R package version 4.0-1. Available: http://CRAN.R-project.org/package=Hmisc. (2016).

[b48] Team R. C. R.: A Language and environment for statistical computing. R Foundation for Statistical Computing, Vienna, Austria. Available: http://www.R-project.org/. (2016).

[b49] Wang Y. . Spatial niche of the rodents in summer in Tangjiahe Nature Reserve. *Acta Theriol*. Sinica 25, 39–44 (2005).

[b50] Shehzad W. . Prey preference of snow leopard (*Panthera uncia*) in South Gobi, Mongolia. PLoS ONE 7, e32104 (2012). 22393381 10.1371/journal.pone.0032104 PMC3290533

[b51] Remonti L., Balestrieri A. & Prigioni C. Altitudinal gradient of Eurasian otter (*Lutra lutra*) food niche in Mediterranean habitats. Can. J. Zool. 87, 285–291 (2009).

[b52] Green K. Altitudinal and temporal differences in the food of foxes (*Vulpes vulpes*) at alpine and subalpine altitudes in the Snowy Mountains. Wildlife Research 30, 245–253 (2003).

[b53] Wang Y., Wang X., Hu J. & Chen L. A preliminary research on small mammal community structure at Tangjiahe Natural Reserve, Sichuan Province, China. *Acta Theriol*. Sinica 23, 39–44 (2003).

[b54] Deagle B. E., Kirkwood R. & Jarman S. N. Analysis of Australian fur seal diet by pyrosequencing prey DNA in faeces. Mol. Ecol. 18, 2022–2038 (2009). 19317847 10.1111/j.1365-294X.2009.04158.x

[b55] Soulé M. E. . Reconstructed dynamics of rapid extinctions of chaparral-requiring birds in urban habitat islands. Conserv. Biol. 2, 75–91 (1988).

[b56] Terborgh J. . Ecological meltdown in predator-free forest fragments. Science 294, 1923–1926 (2001). 11729317 10.1126/science.1064397

[b57] Ma L. Study on the occurrence characteristics of forest pika disaster and its control measures of Mulan Forestry Administration. Hebei Journal of Forestry and Orchard Research 30, 87–89 (2015).

[b58] Li J. & Yang G. Integrated methods for control of forest rodents. Journal of Jilin Forestry Science and Technology 43, 61–62 (2014).

[b59] Paine C. E. T. & Beck H. Seed predation by neotropical rain forest mammals increases diversity in seedling recruitment. Ecology 88, 3076–3087 (2007). 18229842 10.1890/06-1835.1

[b60] Korpimaki E. Rapid or delayed tracking of multiannual vole cycles by avian predators. J. Anim. Ecol. 63, 619–628 (1994).

[b61] Ray J. C. Temporal variation of predation on rodents and shrews by small African forest carnivores. J. Zool. 244, 363–370 (1998).

[b62] Smith A. T. & Foggin J. M. The plateau pika (*Ochotona curzoniae*) is a keystone species for biodiversity on the Tibetan plateau. Anim. Conserv. 2, 235–240 (1999).

[b63] Delibes-Mateos M., Smith A. T., Slobodchikoff C. N. & Swenson J. E. The paradox of keystone species persecuted as pests: A call for the conservation of abundant small mammals in their native range. Biol. Conserv. 144, 1335–1346 (2011).

[b64] Wilson M. C. & Smith A. T. The pika and the watershed: The impact of small mammal poisoning on the ecohydrology of the Qinghai-Tibetan Plateau. AMBIO 44, 16–22 (2015). 25331028 10.1007/s13280-014-0568-x PMC4293360

[b65] Schmitz O. J. Predator diversity and trophic interactions. Ecology 88, 2415–2426 (2007). 18027743 10.1890/06-0937.1

[b66] Bu H. Niche relationships and exploration on coexistence mechanisms of sympatric mesocarnivores in Minshan Mountains, Southwest China Ph.D. thesis, Peking University (2016).

