# Supplementary material for: Molecular dietary analysis of two sympatric felids in the Mountains of Southwest China biodiversity hotspot and conservation implications
Source: Sci Rep. 2017 Feb 14;7:41909. doi: 10.1038/srep41909 (PMC5307313; doi:10.1038/srep41909)

**Molecular dietary analysis of two sympatric felids in the Mountains of Southwest China biodiversity hotspot and conservation implications**

Mengyin Xiong, Dajun Wang, Hongliang Bu, Xinning Shao, Dan Zhang, Sheng Li, Rongjiang Wang and Meng Yao*

School of Life Sciences, Peking University, Beijing 100871, China

* Corresponding author email: yaom@pku.edu.cn

**Appendix S1**

Comparison of the PCR amplification of seven DNA extractions of Asiatic golden cat faecal samples with 12SV5 primers without (on the left) and with (on the right) the blocking oligonulceotide PrioB. PCR products were cloned into a sequencing vector and five independent clones were sequenced for each PCR. Each horizontal bar represents the sequencing results of a faecal DNA sample, and the different colours represent the proportions of clones containing the sequences of the predator or prey items in the sample.

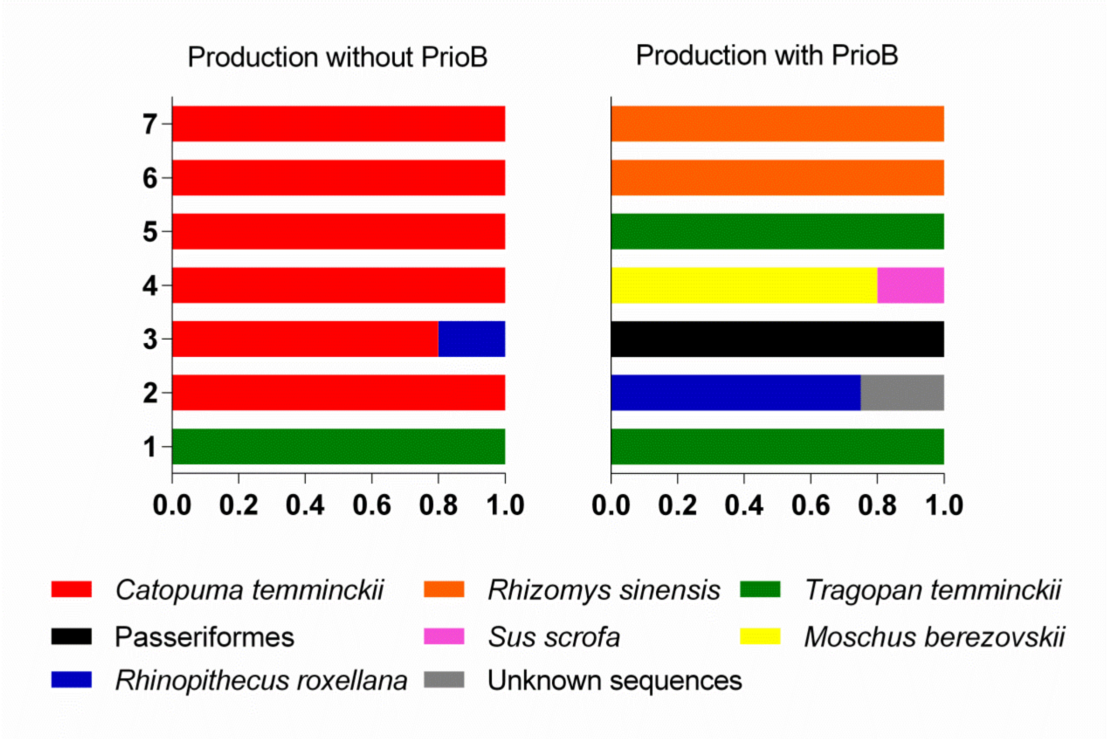

**Appendix S2**

Sequence alignment of the PrioB blocking oligonucleotide with sequences of the leopard cat and Asiatic golden cat and representative vertebrate species commonly occurring in the study area (pika: *Ochotona curzoniae*; rodents: *Niviventer confucianus*, *Apodemus draco* and *Rhizomys sinensis*; shrew: *Episoriculus macrurus*; primate: *Rhinopithecus roxellana*; ungulates: *Sus scrofa*, *Budorcas taxicolor* and *Elaphodus cephalophus*; birds: *Garrulax ocellatus* and *Tragopan temminckii*). Dots (.) indicate identical nucleotides as PrioB; dashes (–) indicate gaps in the sequence compared to PrioB.

| Species | Accession no. | Sequences (5'–3') |
|---|---|---|
| PrioB | | CTATGCTTAGCCCTAAACTTAGATAGTTAATTTTAACAAAACTATC |
| *Prionailurus bengalensis* | JN392459.1 | .............................................. |
| *Catopuma temminckii* | KR132594.1 | ............T...................CA............ |
| *Ochotona curzoniae* | KM225729.1 | ......C.....A.....C..........-CACA-..........T |
| *Niviventer confucianus* | KJ152220.1 | ............T.....C..A...A...-AACCT......A...T |
| *Apodemus draco* | HQ333255.1 | ............T.....C.CA...A..T-GACA-......T...T |
| *Rhizomys sinensis* | AF326254.1 | ............G.....C..AG..A..C-...........T..CT |
| *Episoriculus macrurus* | GU981048.1 | .......C..........C..AG..A..--...A.......T..C. |
| *Rhinopithecus roxellana* | JQ821835.1 | ............T.......TA.....C.-AAAC.....G.....T |
| *Sus scrofa* | KM275217.1 | ......C...........CC.A.......-CA.A-..........T |
| *Budorcas taxicolor* | FJ006534.1 | ..................A..A...A..G-CAAA.....G.T...T |
| *Elaphodus cephalophus* | AY184436.1 | ......C.....T.....AC.A...A..T-CA.A.......T...T |
| *Garrulax ocellatus* | AF484898.1 | ......C.G........TC.T...GC.CG-A.C....CGG.GC... |
| *Tragopan temminckii* | FJ752427.1 | ......C..........TCC.....CCC---.CCT..CT.CG.... |

**Appendix S3**

The equations for calculating dietary parameters.

Dietary specialisation was estimated by the standardised Levins' measure of niche breadth ($B_A$)[1] at the taxon level:

$$B = \frac{1}{\sum p_i^2} \qquad (1)$$

Standardised as: $B_A = \frac{B-1}{n-1}$ (2)

where $B$ is Levins' measure of niche breadth[2], $p_i$ is the percent frequency of occurrence of the $i$th food resource (i.e. %$TX_i$) and $n$ is the total number of resources in the diet.

Dietary diversity was also measured by Shannon's diversity index ($H$) at the taxon level:

$$H = -\sum p_i Ln(p_i) \qquad (3)$$

Evenness of diet was estimated with Peilou's $J$ at the taxon level:

$$J = \frac{H}{\ln(n)} \qquad (4)$$

We used Pianka's measure of niche overlap[3] to estimate dietary overlap between spring (March–May) and autumn (September–November) for the LPC diet at the taxon level:

$$O_{jk} = \frac{\sum_i^n p_{ij} p_{ik}}{\sqrt{\sum_i^n p_{ij}^2 \sum_i^n p_{ik}^2}} \qquad (5)$$

where $O_{jk}$ is Pianka's measure of niche overlap between species $j$ and $k$, $p_{ij}$ is the proportion of the $i$th resource of the total resources used by species $j$ (i.e. %$TX_{ij}$), $p_{ik}$ is the proportion of the $i$th resource of the total resources used by species $k$ (i.e. %$TX_{ik}$) and $n$ is the total number of resource states (i.e. total number of prey taxa). We used

the software EcoSim version 7.72 (http://www.garyentsminger.com/ecosim/) to test whether niche overlap was greater than expected by chance by generating 10,000 simulated matrices of a randomised diet composition.

## References


1 Hurlbert, S. H. The measurement of niche overlap and some relatives. *Ecology* **59**, 67-77 (1978).

2 Levins, R. *Evolution in changing environments: some theoretical explorations*. (Princeton University Press, 1968).

3 Pianka, E. R. The structure of lizard communities. *Annu. Rev. Ecol. Syst.* **4**, 53-74 (1973).

**Appendix S4**

Altitudinal variations in diet composition by the prey orders identified in the leopard cat diet in northern Sichuan, China. < 1,500 m: *n* = 15; 1,500–2,000 m: *n* = 32; 2,000–2,500 m: *n* = 31; > 2,500 m: *n* = 13. Diet composition is presented as a percent frequency of occurrence (%FC).

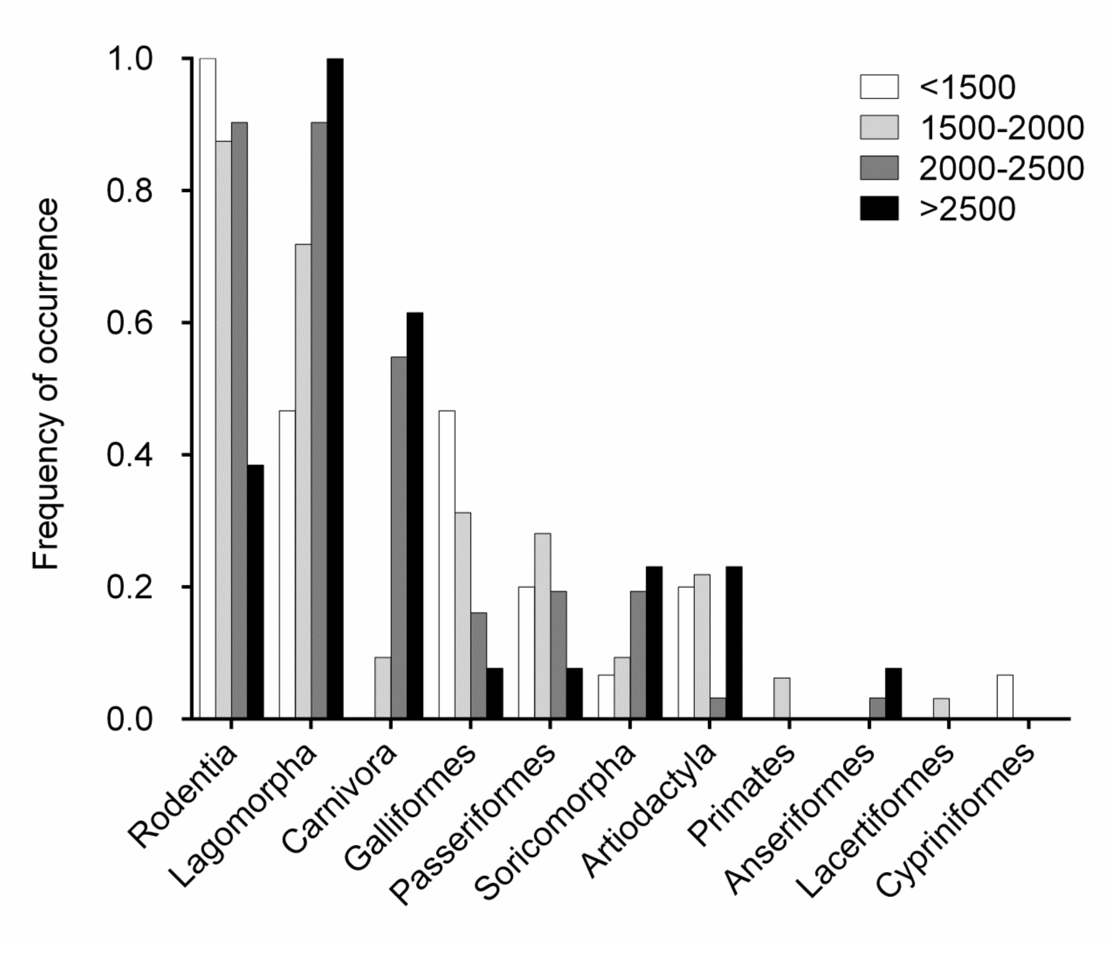

Supplement: Supplementary Appdendex 1-4 [file srep41909-s1.pdf]
